# Examining Public Perceptions about Lead in School Drinking Water: A Mixed-Methods Analysis of Twitter Response to an Environmental Health Hazard

**DOI:** 10.3390/ijerph15010162

**Published:** 2018-01-20

**Authors:** Christine C. Ekenga, Cora-Ann McElwain, Nadav Sprague

**Affiliations:** 1 Brown School, Washington University in St. Louis, St. Louis, MO 63130, USA; c.mcelwain@wustl.edu; 2 Gateway to the Great Outdoors, Chicago, IL 60613, USA; nadav@gatewayoutdoors.org

**Keywords:** lead, public health, environmental exposure, inequalities, social media, thematic analysis

## Abstract

Exposure to lead has long been a community health concern in St. Louis, Missouri. The objective of this study was to examine public response to reports of elevated lead levels in school drinking water in St. Louis, Missouri via Twitter, a microblogging platform with over 320 million active users. We used a mixed-methods design to examine Twitter user status updates, known as “tweets,” from 18 August to 31 December 2016. The number of tweets each day was recorded, and Twitter users were classified into five user types (General Public, Journalist/News, Health Professional/Academic, Politician/Government Official, and Non-Governmental Organization). A total of 492 tweets were identified during the study period. The majority of discourse on Twitter occurred during the two-week period after initial media reports and was driven by members of the General Public. Thematic analysis of tweets revealed four themes: Information Sharing, Health Concerns, Sociodemographic Disparities, and Outrage. Twitter users characterized lead in school drinking water as an issue of environmental inequity. The findings of this study provide evidence that social media platforms can be utilized as valuable tools for public health researchers and practitioners to gauge public sentiment about environmental health issues, identify emerging community concerns, and inform future communication and research strategies regarding environmental health hazards.

## 1. Introduction

Exposure to lead through drinking water is a public health hazard, particularly for children [1,2]. Lead is a neurotoxin and elevated blood lead levels in children have been associated with a range of adverse health effects, including learning disabilities, behavioral problems, and reduced cognitive function [3,4,5,6,7]. In the United States (US), blood lead levels among children have declined [8,9], particularly since the 1970s, when lead-based paint was banned, leaded gasoline was phased out, and many public health agencies introduced lead prevention initiatives. Nevertheless, differences in the blood lead levels among US children persist, with children living in urban settings with aging infrastructure being among the most vulnerable [9].

Recent, high profile reports of lead in drinking water have led to an elevated awareness of this public health hazard [10]. The Flint, Michigan water crisis, for example, placed a spotlight on leaded pipes that were installed long before the banning of these pipes from public water systems and plumbing. The failure of public officials to treat water with corrosion inhibitors after a water source change led to the leaching of lead into the city’s water supply, potentially exposing residents to elevated levels of lead in drinking water [11]. After this change in water source, the percentage of Flint children with elevated blood lead levels increased, garnering widespread attention from the media and igniting public outcry worldwide [12,13].

In the aftermath of the Flint water crisis, social media was one of the tools used by the public, at large, to express concern about lead-contaminated drinking water. As the public sought more information about the crisis, Twitter hashtags, such as #FlintWaterCrisis, facilitated communication between users and across communities [14]. In recent years, social media has expanded avenues for public opinion and information sharing. Additionally, social media has changed the way the public consumes health information. Approximately 62% of US adults receive their news from social media sites [15], and studies have shown that social media users are more likely to use these platforms than traditional media to communicate with friends and family about a crisis than traditional media sources [16]. 

Analysis of social media communications have the potential to provide useful information about public awareness, reaction, and opinion of emerging public health crises. A key aspect of public health preparedness and response involves the investigation of new and emerging technologies to monitor health outcomes, promote public health awareness, and engage communities in hazard response and recovery. In the case of environmental health hazards, studying social media can provide public health practitioners and researchers with new opportunities for surveillance before, during, and after an environmental health crisis. Recent examples include studies of public sentiment and discourse about the Zika virus [17], the 2014 Ebola outbreak [18], and environmental disasters in the United States such as the 2010 Deepwater Horizon oil spill [19] and Hurricane Sandy [20]. In fact, Twitter, a microblogging platform with over 320 million active users [21], has been mined to examine a variety of health-related topics, from mental health discourse to infectious disease outbreaks [22,23,24]. 

Amid media coverage about drinking water quality, school districts across the US initiated testing of drinking water supplies for lead, including the St. Louis Public Schools (SLPS) in St. Louis, Missouri. SLPS is the largest public school district in the St. Louis metropolitan area, one of the most segregated metropolitan regions in the United States [25]. In 2016, elevated (>10 parts per billion (ppb)) levels of lead were reported to be present in the drinking water sources of SLPS buildings [26]. Exposure to lead has long been an ongoing health concern for residents of the City of St. Louis [27,28]. Thus, the objective of this study was to use Twitter to examine the public response to reports of elevated lead levels in school drinking water in St. Louis, Missouri. Investigating the public response to this hazard will inform future communication and research strategies regarding environmental health hazards.

## 2. Methods 

### 2.1. Study Context 

In Summer 2016, the SLPS commissioned testing of water samples from drinking fountains and sinks in 74 buildings across the district. Preliminary results indicated that, out of the 744 water sources tested, 82 sources in 30 different buildings had water samples with lead levels above 10 ppb [26]. A total of 16 schools had samples with lead levels above 30 ppb, with the highest level ranging from 200–300 ppb [26]. In response to these test results, the SLPS turned off the water sources at all of the affected fountains and sinks and supplied bottled water to the 12 schools in which elevated levels were observed near cafeteria drinking water sources. In addition, the SLPS mailed letters to the parents of students at all 30 identified schools notifying them about the elevated lead levels. 

On 18 August 2016, the St. Louis Post Dispatch, the only printed daily newspaper in the St. Louis metropolitan area, reported on the elevated lead levels in SLPS drinking water sources [26]. One week later, the SLPS reported the lead testing results to the public at a Board of Education meeting, where the Board approved more than one million dollars to replace pipes, sinks, and drinking fountains in the affected schools [29]. Details of the testing results were subsequently reported throughout the local news media, initiating strong public response, on and off Twitter, from a broad range of individuals and organizations.

### 2.2. Search Strategy

A cross-sectional study of public perceptions was conducted using content analysis of Twitter status updates, also known as tweets. The Twitter application programming interface (API) function was used to identify tweets about SLPS lead testing-related results between 18 August 2016, the date of the initial media report of elevated lead level in drinking water source sample, and 31 December 2016. 31 December was chosen as the end date to allow the capture of tweets up to three weeks after 8 December 2016, the date of the last SPLS Board of Education report to the public regarding the August 2016 lead testing results. Searches included the following keywords: “lead,” “water,” “schools,” “St. Louis,” and the names of the 30 schools where elevated lead levels were found.

### 2.3. Quantitative Analysis

The number of tweets each day was recorded, and we determined the total number of original tweets, retweets (reposting of another user’s tweet), and replies (to an original tweet or retweet) during the study period. We also classified Twitter users into five user types (General Public, Journalist/News, Health Professional/Academic, Politician/Government Official, and Non-Governmental Organization). User types were identified based on a review of the Twitter user’s profile page and biography. Two independent reviewers coded each user profile. We used the Kappa statistic to evaluate agreement between the two coders. Briefly, the Kappa statistic is a measure of agreement between two observers that takes chance into account. Scores range from −1.0 to 1.0, with 1.0 indicating perfect agreement, 0 indicating completely random agreement, and −1.0 indicating perfect disagreement [30]. According to Landis and Koch’s recommendations [31], Kappa scores from 0.0 to 0.2 indicate slight agreement, 0.21 to 0.40 indicate fair agreement, 0.41 to 0.60 indicate moderate agreement, 0.61 to 0.80 indicate substantial agreement, and 0.81 to 1.0 indicate strong agreement. Interrater reliability for each user type ranged from Kappa = 0.85 to Kappa = 0.96. Descriptive statistics were summarized in terms of counts and percentages by user type. All statistical analyses were performed using the Statistical Package for the Social Sciences software, version 24.0 (SPSS Inc., Chicago, IL, USA).

### 2.4. Qualitative Analysis

We conducted a thematic analysis of tweet texts using Braun and Clarke’s method [32]. Briefly, we sought to identify themes as they emerged during a review of the data rather than an analysis based on a pre-existing theoretical framework. First, two trained reviewers conduct an initial review to familiarize themselves with the tweets. Next, the reviewers reviewed a subset of tweets independently and generated a preliminary set of codes. The codes were characterized according to their relationships and linked and grouped into meaningful themes. Potential themes were then evaluated against another subset of data and refined as the review progressed through all of the tweets. After reviewing and grouping codes for all tweets, final themes were defined, and representative tweets for each theme were selected. When necessary, tweets containing multiple themes were coded with more than one theme. Interrater reliability for each theme ranged from Kappa = 0.78 to Kappa = 0.96. We also coded the sentiment of each tweet as positive (expressing positive emotions or praise), negative (expressing negative emotions or criticism), and neutral.

## 3. Results

### 3.1. Quantitative Results

**Figure 1:**
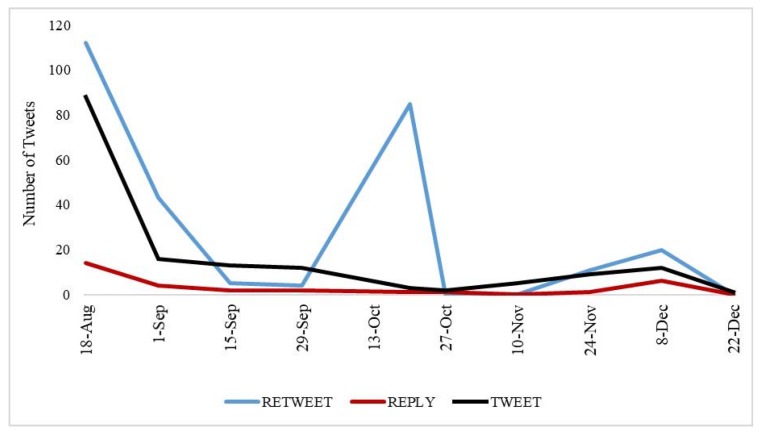
Number of tweets during the study period (18 August–31 December 2016).

During the 135-day study period, there was a total of 492 unique tweets by 286 Twitter users. Approximately 33% of these tweets were original tweets, 61% were retweets, and 6% were replies. The highest number of total tweets (129) occurred on 18 August 2016, the date of the first news report of elevated lead levels in SLPS drinking water sources. The two-week period immediately following this initial report (19–31 August 2016) averaged 15 tweets per day. The greatest number of retweets (85) occurred on 20 October 2016, after a variety of news outlets reported on the October SPLS Board of Education meeting, which included an announcement that replacement pipes would not be installed until November 2016, weeks after initial target dates [25]. The number of tweets dropped dramatically after this second peak, resulting in a total of 60 tweets during the months of November and December (Figure 1).

Analyses of the 243 available (85%) Twitter user profiles and biographies revealed that members of the General Public accounted for 63% of all tweets, followed by Journalists/News Organizations (18%), Health Professionals/Academics (14%), Politician/Government Officials (4%), and Non-Governmental Organizations (1%). Approximately 72% (190/264) of tweets by members of the General Public were replies or retweets, while 84% (62/74) of tweets by Journalists or News Organizations were original tweets (Table 1).

### 3.2. Qualitative Results

**Figure 2:**
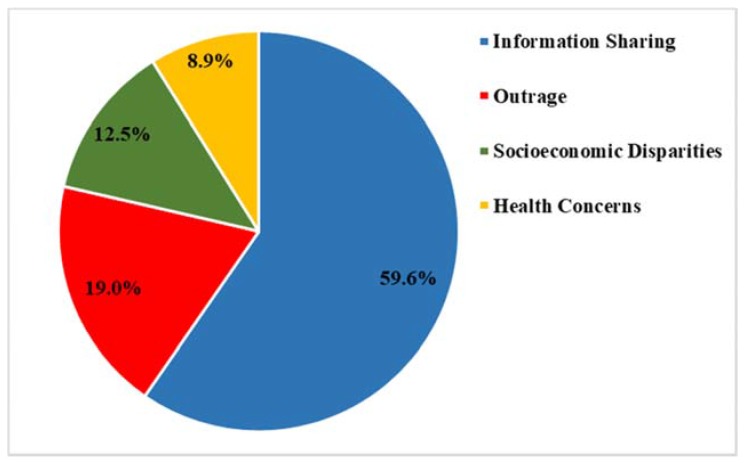
Thematic categories.

Thematic analysis resulted in a total of four common thematic categories: Information Sharing (59.6% of all tweets), Health Concerns (8.9%), Sociodemographic Disparities (12.5%), and Outrage (19%) (Figure 2). 

Table 2 displays each theme with representative statements. Sentiment analysis revealed that most (74%) opinion-based tweets were negative in sentiment, with 21% expressing neutral sentiment, and 5% expressing positive sentiment (Table 3).

#### 3.2.1. Information Sharing

The majority of these tweets, including retweets and replies, in this theme were characterized by sharing articles and information on the status of the lead found in SLPS water fountains. Many Twitter users shared links to news articles or podcasts that explained the situation in greater detail. Most tweets did not include the Twitter user’s opinion on the topic. However, some Twitter users applauded SLPS for the actions being taken to remedy the situation while others criticized SLPS for being “dysfunctional” and for not responding to the elevated water lead levels in an appropriate manner.

#### 3.2.2. Health Concerns

Twitter users commented on the potential health impacts of lead in school drinking water. The strongest sentiments expressed by Twitter users described the “toxic” and “poisonous” lead as being at “dangerous” levels for children. Additionally, some users referred to well-established associations between elevated levels of lead, violent behavior, cancer, and adverse physical and mental health outcomes, particularly in children. 

#### 3.2.3. Sociodemographic Disparities

Tweets under the Socioeconomic Disparity theme emphasized concerns about environmental inequities in the St. Louis metropolitan region. Users highlighted socioeconomic and racial disparities among school districts in the region. Some users made unfavorable comparisons between the inaction of poor school districts and the immediate actions undertaken in school districts in more affluent areas. Media reports of prompt removal and replacement of lead-tainted drinking water sources in predominately White school districts were highlighted in these tweets. Several users connected these disparities to environmental racism, highlighting the disproportionate exposures and responses to lead in school drinking water in predominately African-American school districts throughout the region.

#### 3.2.4. Outrage

Original tweets and retweets categorized under the Outrage theme indicate that the Twitter users were dismayed by the discovery of high lead levels in St. Louis public school water, the danger that lead poses to children, and the inadequate response from school and local government officials. 

Emotions conveyed by the tweets sparked original tweet/retweet exchanges about the quality and effectiveness of the school district. Some Twitter users expressed that the high lead levels in public school water could be a result of deficient public school funding, while others vehemently supported school vouchers that would allow the children at the affected schools to attend school where they can safely drink the water. Numerous Twitter users used the social media platform to publicly demand action, whether directly or indirectly, from school and government officials.

Many of the tweets in this theme overlapped the Sociodemographic Disparity theme because Twitter users were emotionally dismayed that the public schools most affected by high levels of lead were the schools with a majority populations of African-American and low-income children. Some Twitter users vehemently communicated their belief that the lead found in SLPS drinking water was present at levels unacceptable to human health.

## 4. Discussion

The study provides quantitative and qualitative evidence data on public response to media reports of elevated lead levels in school drinking water in St. Louis, Missouri. Using analysis of Twitter user status updates (tweets), we observed that the majority of discourse on Twitter occurred during the two-week period after initial reports and was driven by concerned members of the general public. In addition, thematic analysis revealed that Twitter users perceived drinking water contamination in St. Louis Public School to be a social justice issue as well as an environmental health issue. The results of this study suggest that Twitter users characterize lead in school drinking water in St. Louis, Missouri as an issue of environmental inequity.

Childhood lead poisoning has been an ongoing public health concern in St. Louis, Missouri. Children in St. Louis make up approximately 40% of elevated blood lead level results in Missouri, while accounting for only 14% of Missouri’s lead-tested population [27]. In response to these rates, the City of St. Louis implemented a lead prevention program that provides case management, awareness and prevention activities, and environmental risk assessments to the community [28]. These efforts, in addition to mandated lead screening for children in at-risk districts, have been successful in decreasing the frequency of lead poisoning in St. Louis from 1 in 4 children in the 1990s to 1 in 50 children in 2011 [28]. Nevertheless, rates of elevated lead blood levels in the City of St. Louis remain among the highest in the state [28]. This historical context, coupled with the events of the Flint, Michigan water crisis, produced renewed concerns about lead as community health hazard in St. Louis; this time regarding the presence of lead in school drinking water.

Missouri’s history in lead exploration and its aging infrastructure makes the state’s residents, especially residents of concentrated urban areas like St. Louis, at risk for elevated exposure to lead. Elevated levels of lead are most commonly found in buildings that were constructed prior to the 1980s or in buildings that have not been renovated since the 1980s [28,29]. In wealthier school districts in St. Louis metropolitan region, there were relatively fewer reports of elevated levels of lead in school drinking water [33], and these school districts’ more robust budget allowed the districts to regularly test for lead in the drinking water, replace problematic water sources, and build new schools more frequently [33]. In contrast, for the St. Louis Public Schools, where the student population included a higher percentages of minority children [33], approximately 11% of the water sources in school buildings yielded positive results for elevated lead levels [26]. 

Our findings provide information that can inform future environmental health research efforts in the St. Louis Metropolitan area. For example, we observed that reported disparities in school drinking water quality elicited strong responses from Twitter users. Therefore, communication strategies should account for, and be sensitive to, sociodemographic differences in environmental exposures. Our findings have implications for community engagement as well. In the event of future environmental public health emergencies and disasters, social medial platforms such as Twitter can serve as a tool for both communication and surveillance for affected community members, public health practitioners, and public health researchers.

Our study identified concerns about lead in school drinking water in the St. Louis metropolitan region. However, our analyses are not without limitations. First, because Twitter API sampling is capped at approximately 1% of all Tweets at a given time [34], it is likely that the current study underestimated the total number of tweets about elevated lead levels in SLPS drinking water sources during our study period. Second, although we used search terms specific to the SLPS elevated lead results, (e.g., “St. Louis,” “Saint Louis,” and the names of schools where elevated lead levels were observed), the Twitter API does not have a function to filter tweets by geographic location. Thus, we could not confirm if public response on Twitter was representative of the public response in the greater St. Louis metropolitan area. Third, publically available tweets may not be representative of the sentiments of Twitter users with private accounts or the general population at large. In fact, user demographics indicate that Twitter users tend to be younger than the general population [35]. Lastly, Twitter user types may have been misclassified because of the available information during profile reviews. Although two study investigators independently reviewed each Twitter user’s profile page and biography for demographic information, the characterizations of Twitter user types could not independently be verified.

## 5. Conclusions

Analysis of Twitter user sentiment after reports of elevated lead levels in St. Louis Public School drinking water revealed a mix of concern and outrage about environmental inequities in the St. Louis metropolitan region. As this study is a first step in examining public perceptions of environmental health hazards using social media data, future research should incorporate and compare data from other social networking sites, such as Facebook. Given the growing presence and increased influence of social media, these platforms have the potential to provide opportunities for public health researchers and practitioners to gauge public sentiment about environmental health issues, identify emerging community concerns, and inform future communication and research strategies regarding environmental health hazards.

## Figures and Tables

**Table 1:** Characteristics of tweets by users with publically available profiles.

User Type	Total	Original	Retweet	Reply	No. of Followers
	*n* (%)	*n* (%)	*n* (%)	*n* (%)	Mean (SD)
General Public	264 (62.9)	51 (37.5)	190 (75.4)	23 (71.9)	273 (193)
Journalist/News Organization	74 (17.6)	62 (45.6)	9 (3.6)	3 (9.4)	110,054 (51,329)
Health Professional/Academic	59 (14.0)	13 (9.6)	43 (17.1)	3 (9.4)	99 (70)
Politician/Government Official	18 (4.3)	8 (5.9)	7 (2.8)	3 (9.4)	32,785 (12,982)
Non-Governmental Organization	5 (1.2)	2 (1.5)	3 (1.2)	0 (0)	777 (514)

**Table 2:** Example tweets by theme.

**Information Sharing**
Lead level almost 20 times benchmark among 32 St. Louis school buildings with elevated levels
Public schools in St. Louis take action against lead found in drinking water
Elevated Lead Levels Found in 30 St. Louis School Buildings
St. Louis Public School Board approves $1-million for lead remediation in water in schools
**Health Concerns**
Chicago. St Louis, etc. have lead in water. Lead causes violent behavior.
If the #moleg continues to be gripped by dysfunction, the health & safety of MO children could be the cost.
Understanding the dangers of lead contamination in school fountains
Lead Levels Risk to childrens health, @stltodays Report on Lead levels in water of St. Louis schools
**Sociodemographic Disparities**
Lead Poisoning Is Higher In St. Louis Than Flint Michigan Its not about Flint or water, it’s about race inequity in America
St Louis: Where black kids go to school w/lead contaminated water fountains that are decades old.
In poor black schools in St, Louis, water fountains poisoned with lead. In rich white schools, they’re gold-plated.
Toxic Lead in School Water Fountains, A Symbol of St. Louis inequality: Clean donor-funded water for white & wealthy
Lead found in St. Louis schools is just the latest link in a generations-long struggle against environmental racism.
And they stay shortchanging black children. their solution to the lead in the water epidemic in St. Louis.
**Outrage**
So there is dangerous amounts of lead in the drinking water at St Louis Public schools...officials say it may be WEEKS before its removed..
Another reason funding public schools should ALWAYS be a top priority. Fix the pipes!
Lead in St. Louis schools twice the federal threshold. How can we be educating our kids, & poisoning at same time??
Stay in school they said, but bring your own water because our school’s is filled with lead. St. Louis, rise up.

**Table 3:** Sentiment of tweets.

Sentiment	Total Tweets (*n* = 247) *
	*n* (%)
Negative	182 (73.7)
Neutral	52 (21.0)
Positive	13 (5.3)

* Sentiment of tweet was determined only for opinion-based tweets.
